# Activation of the orphan receptor GPR55 by lysophosphatidylinositol promotes metastasis in triple-negative breast cancer

**DOI:** 10.18632/oncotarget.10206

**Published:** 2016-06-21

**Authors:** Clara Andradas, Sandra Blasco-Benito, Sonia Castillo-Lluva, Patricia Dillenburg-Pilla, Rebeca Diez-Alarcia, Alba Juanes-García, Elena García-Taboada, Rodrigo Hernando-Llorente, Joaquim Soriano, Sigrid Hamann, Antonia Wenners, Ibrahim Alkatout, Wolfram Klapper, Christoph Rocken, Maret Bauer, Norbert Arnold, Miguel Quintanilla, Diego Megías, Miguel Vicente-Manzanares, Leyre Urigüen, J. Silvio Gutkind, Manuel Guzmán, Eduardo Pérez-Gómez, Cristina Sánchez

**Affiliations:** ^1^ Department of Biochemistry and Molecular Biology I, School of Biology, Complutense University, Madrid, Spain; ^2^ Instituto de Investigación Hospital 12 de Octubre, Madrid, Spain; ^3^ Oral and Pharyngeal Cancer Branch, National Institute of Dental and Craniofacial Research, National Institutes of Health, Bethesda, MD, USA; ^4^ Department of Pharmacology, University of The Basque Country UPV/EHU, and Centro de Investigación Biomédica en Red de Salud Mental (CIBERSAM), Spain; ^5^ Instituto de Investigación Sanitaria Hospital Universitario de la Princesa and Universidad Autónoma de Madrid, School of Medicine, Madrid, Spain; ^6^ Spanish National Cancer Research Centre (CNIO), Madrid, Spain; ^7^ Department of Gynecology and Obstetrics, University Hospital Schleswig-Holstein, Kiel, Germany; ^8^ Institute of Pathology, University Hospital Schleswig-Holstein, Kiel, Germany; ^9^ Instituto de Investigaciones Biomédicas Alberto Sols, Consejo Superior de Investigaciones Científicas-Universidad Autónoma de Madrid, Madrid, Spain; ^10^ Department of Pharmacology, University of California San Diego, Moores Cancer Center, La Jolla, CA, USA; ^11^ Centro de Investigación Biomédica en Red de Enfermedades Neurodegenerativas (CIBERNED) and Instituto Ramón y Cajal de Investigación Sanitaria (IRYCIS), Madrid, Spain

**Keywords:** GPR55, G protein-coupled receptor, cannabinoids, metastasis, triple-negative breast cancer

## Abstract

The orphan G protein-coupled receptor GPR55 has been directly or indirectly related to basic alterations that drive malignant growth: uncontrolled cancer cell proliferation, sustained angiogenesis, and cancer cell adhesion and migration. However, little is known about the involvement of this receptor in metastasis. Here, we show that elevated GPR55 expression in human tumors is associated with the aggressive basal/triple-negative breast cancer population, higher probability to develop metastases, and therefore poor patient prognosis. Activation of GPR55 by its proposed endogenous ligand lysophosphatidylinositol confers pro-invasive features on breast cancer cells both *in vitro* and *in vivo*. Specifically, this effect is elicited by coupling to G_q/11_ heterotrimeric proteins and the subsequent activation, through ERK, of the transcription factor ETV4/PEA3. Together, these data show that GPR55 promotes breast cancer metastasis, and supports the notion that this orphan receptor may constitute a new therapeutic target and potential biomarker in the highly aggressive triple-negative subtype.

## INTRODUCTION

G protein-coupled receptors (GPCRs), the largest superfamily of receptors, are involved in a wide variety of biological functions [1], and their dysfunction contributes to many human diseases [2]. Increasing evidence indicates that aberrant GPCR signaling is implicated in cancer initiation and progression [3–5], and therefore the search for new GPCRs involved in these processes has become a strategy for the development of new cancer treatments. GPR55 is a GPCR that is engaged by lipids, specifically lysophosphatidylinositol (LPI) and certain cannabinoid compounds [6–8]. However, due to the lack of proof for *in vivo* activation by these ligands, this receptor remains in the Class A orphan subfamily [9]. Increasing evidence supports that GPR55 is an important component of the molecular circuitry that controls cancer cell behavior. Thus, this receptor has been shown to drive cancer cell proliferation in *in vitro* and/or *in vivo* models of glioblastoma [10]; prostate [11], ovarian [11] and skin carcinoma [12]; melanoma [13], and non-small lung cancer [14]. GPR55 has also been indirectly associated to both pro-angiogenic responses [15] and to pro-migratory phenotypes in breast [16] and colon cancer cells [17]. However, little is known about the real impact of these effects on the metastatic process, the final and most lethal stage of cancer progression, as well as the molecular mechanisms governing those actions. Hence, here we aimed at shedding light on these two particular issues by focusing on breast cancer, one of the leading causes of death in women [18].

## RESULTS

### GPR55 expression correlates with triple-negative tumors and poor patient prognosis

**Figure 1:**
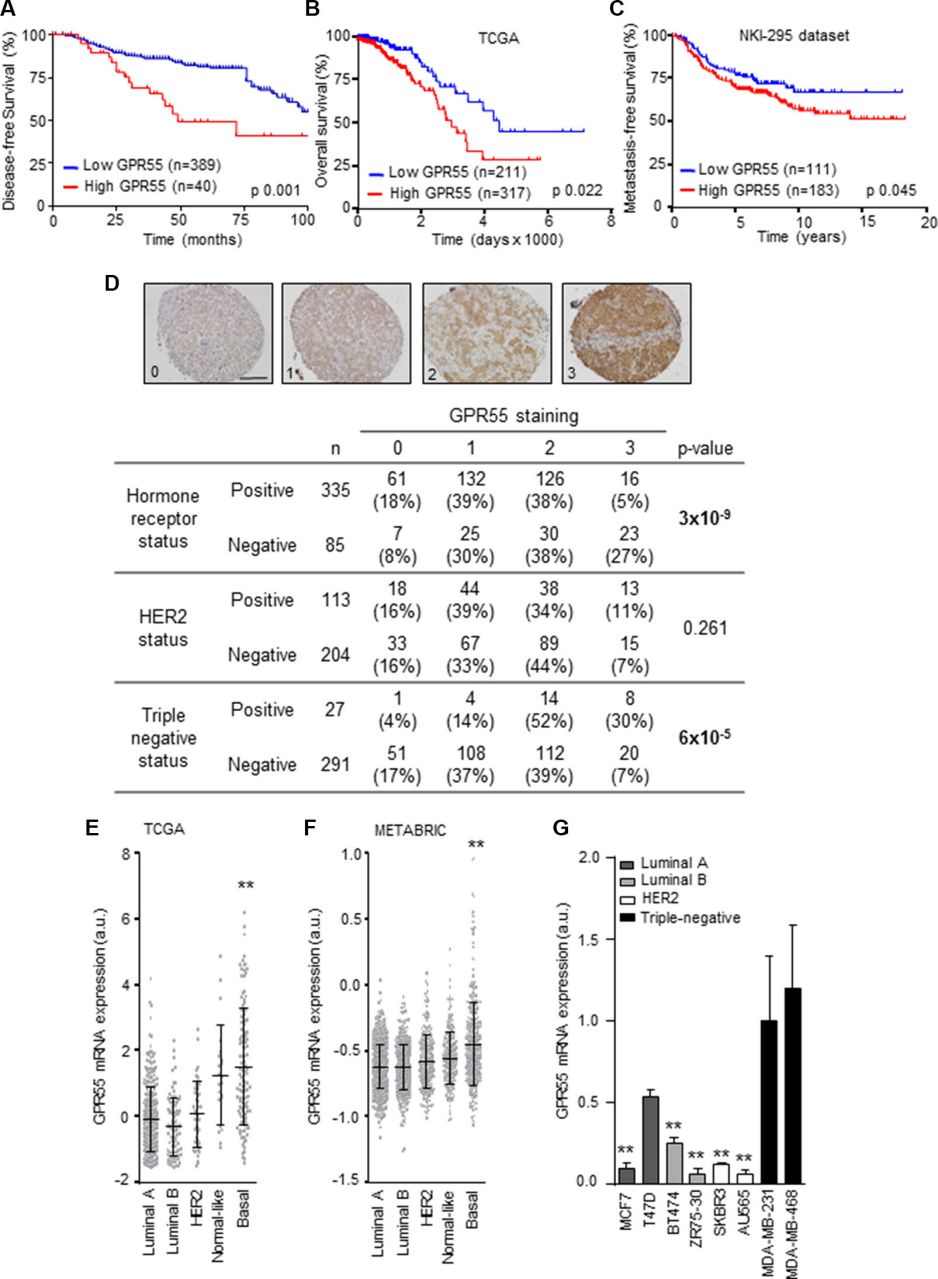
GPR55 expression correlates with triple-negative tumors and poor patient prognosis (**A–C**) Kaplan-Meier curves for disease-free survival (A), overall survival (B), and metastasis-free survival (C). Data plotted in A correspond to the human breast tumor tissues with complete clinical information contained in the 483-sample tissue microarray generated at the University Hospital of Kiel and described in [19]. Cases were scored as 0 (no staining), 1 (weak staining), 2 (moderate staining), or 3 (high staining), for GPR55 expression. A representative image of each category is shown in (**D**) upper panel. Scale bar, 0.25 mm. Samples scoring 0 were grouped as “low GPR55 expression”, and cases scoring 1-3 as “high GPR55 expression”. Data plotted in (B and C) were obtained from the microarray data sets published by the TCGA network in [20] (B), and from the microarray data set published in [21] (C). In these two panels, samples were ranked by GPR55 mRNA expression, and the best cutoff was manually selected. Survival curves were statistically compared by the log-rank test. (D) Association between GPR55 expression (as determined by staining scoring) and the molecular features of the breast tumor samples included in the TMAs described in (A). The Pearson's chi-squared test was used for statistical analysis. (**E–G**) Relative GPR55 mRNA expression in the indicated breast cancer subtypes (E and F) or human breast cancer cell lines (G). Data in (E and F) were obtained from the same database as in (B) (for E), and from database in [22] (in F). **p < 0.01 *vs.* the rest of the subgroups [except Normal-like in (E)], and *vs.* MDA-MB-231 cells in (G).

To understand the role of GPR55 in the advanced stages of breast cancer progression, we first investigated whether there was an association between GPR55 levels and patient prognosis. We analyzed GPR55 protein expression in a tissue microarray (TMA) containing 483 breast human samples [19]. We found a strong correlation between high GPR55 expression and reduced disease-free survival (Figure 1A). To determine whether this association was also observed at the mRNA level, we analyzed the publically available TCGA microarray data set that contains molecular and clinical data from 825 breast cancer patients [20]. Women with high tumor GPR55 mRNA expression presented reduced overall survival than those with low GPR55 mRNA levels (Figure 1B). In an additional dataset containing 295 breast cancer samples [21], high GPR55 mRNA expression was associated with reduced metastasis-free survival (Figure 1C). Since breast cancer is a very heterogeneous disease, we studied whether GPR55 expression was associated to a specific molecular subtype. We found a strong association between GPR55 protein levels and triple-negative tumors (Figure 1D). Specifically, moderate or high GPR55 staining was found in 82% of them (Figure 1D). Likewise, GPR55 mRNA levels were elevated in basal tumors with respect to the other molecular subtypes of breast cancer (i.e. normal-like, luminal A or B, and HER2-enriched) in two datasets containing a total of 2557 human samples [20, 22] (Figures 1E and 1F), and in basal human breast cancer cell lines with respect to cell lines with other molecular features (Figure 1G). Together, these findings show that an elevated GPR55 expression is associated with the highly aggressive basal/triple-negative breast cancer subtype, higher probability to develop metastases, and therefore poor patient prognosis.

### GPR55 bestows pro-metastatic advantages to breast cancer cells *in vitro* and *in vivo*

**Figure 2:**
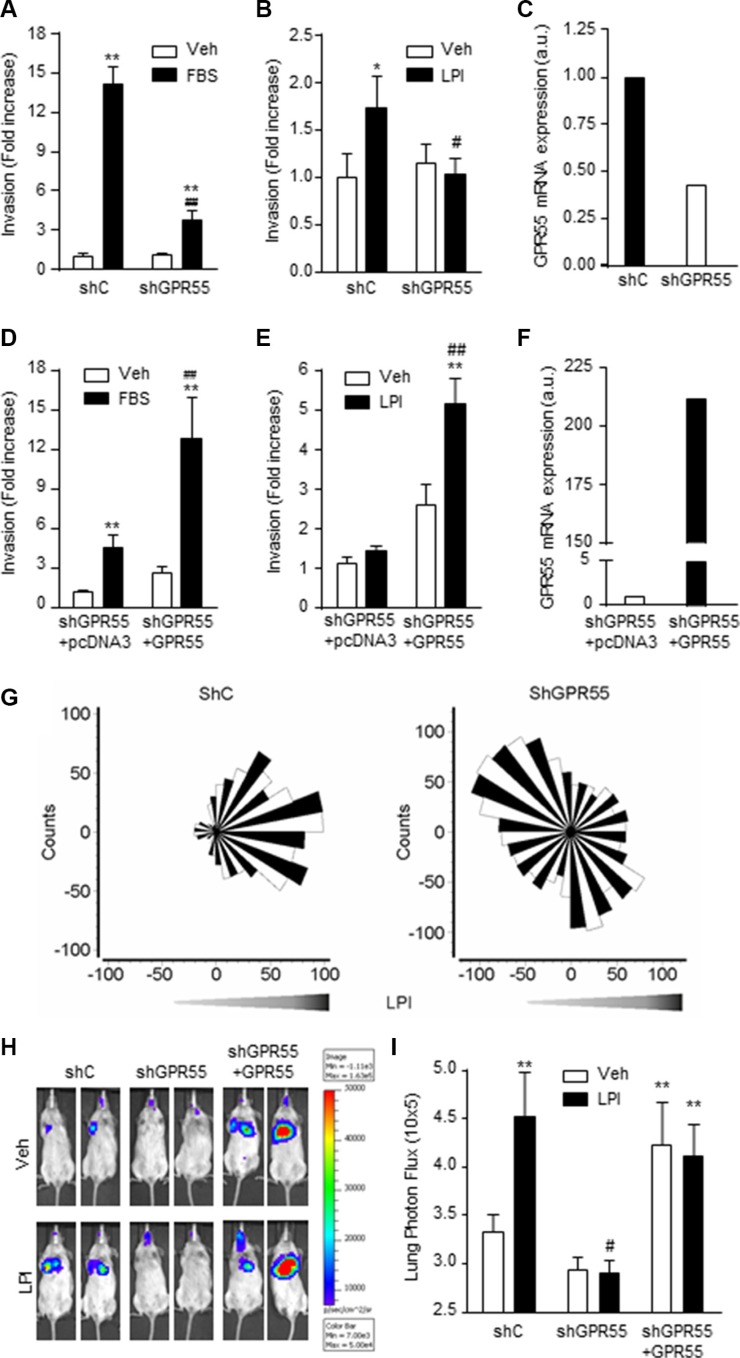
GPR55 confers pro-metastatic advantages on breast cancer cells *in vitro* and *in vivo* (**A–F**) Cell invasion assays were performed with four different cell lines: MDA-MB-231 cells stably expressing a shRNA selectively targeting GPR55 (shGPR55) or a non-targeted shRNA (shC) (A–C), or shGPR55 cells stably expressing a GPR55 overexpression plasmid (shGPR55 + GPR55) or the corresponding empty vector (shGPR55 + pcDNA3) (D–F). Results are expressed as invasion fold increase towards 10% FBS or 0.5 μM LPI *vs.* shC (A and B) or shGPR55 + pcDNA3 (D and E) vehicle-treated cells, set at 1. (C and F) Relative GPR55 mRNA expression in GPR55 knocked-down (C) and GPR55 overexpressing cells (F). (**G**) Angular histograms of shC (left panel) and shGPR55 (right panel) cells migrating in response to a LPI gradient. (**H**) Representative images of the lung metastases generated by injection of the indicated luciferase expressing cell lines. (**I**) Quantification of the lung bioluminescence signal. *p < 0.05; **p < 0.01 *vs.* shC or shGPR55 + pcDNA3 vehicle-treated cells/animals. #*p* < 0.05; ##*p* < 0.01 *vs.* shC or shGPR55 + pcDNA3 FBS- or LPI-treated cells/animals.

As high tumor aggressiveness is intimately related to the capability of cancer cell dissemination and the establishment of metastatic lesions, we next analyzed whether GPR55 bestows pro-metastatic properties to triple-negative breast cancer cells. First, we studied the role of GPR55 in cancer cell invasion, a pivotal event involved in the generation of metastases [23]. Triple-negative MDA-MB-231 breast cancer cells increased their invasive behavior when either FBS (Figure 2A) or LPI (Figure 2B) was used as chemoattractant. This effect was markedly reduced when GPR55 expression was knocked-down (Figures 2A–2C), and rescued upon GPR55 ectopic overexpression (Figures 2D–2F). The role of LPI as a positive directional cue for GPR55 expressing cells was substantiated by directional migration experiments. Thus, the migration towards LPI observed in MDA-MB-231 cells (Forward Migration Index = 0.117) was abolished by GPR55 knockdown (Forward Migration Index = −0.025, Figure 2G). Similar effects were observed in an animal model of lung metastasis. Mice injected with MDA-MB-231 cells expressing endogenous levels of GPR55 bore significantly more metastases when treated with LPI than when treated with vehicle (Figures 2H and 2I). Injection into the circulation of MDA-MB-231 cells with reduced GPR55 expression generated significantly less lung metastases than injection of control cells, and, in this case, LPI did not increase the number of metastatic lesions (Figures 2H and 2I). Moreover, the number of metastases augmented when the injected cells overexpressed GPR55 (Figures 2H and 2I). These observations show that activation of GPR55 bestows pro-metastatic features to breast cancer cells both *in vitro* and *in vivo*.

### GPR55-driven pro-invasive responses involve coupling to G_q/11_ and activation of RhoA

**Figure 3:**
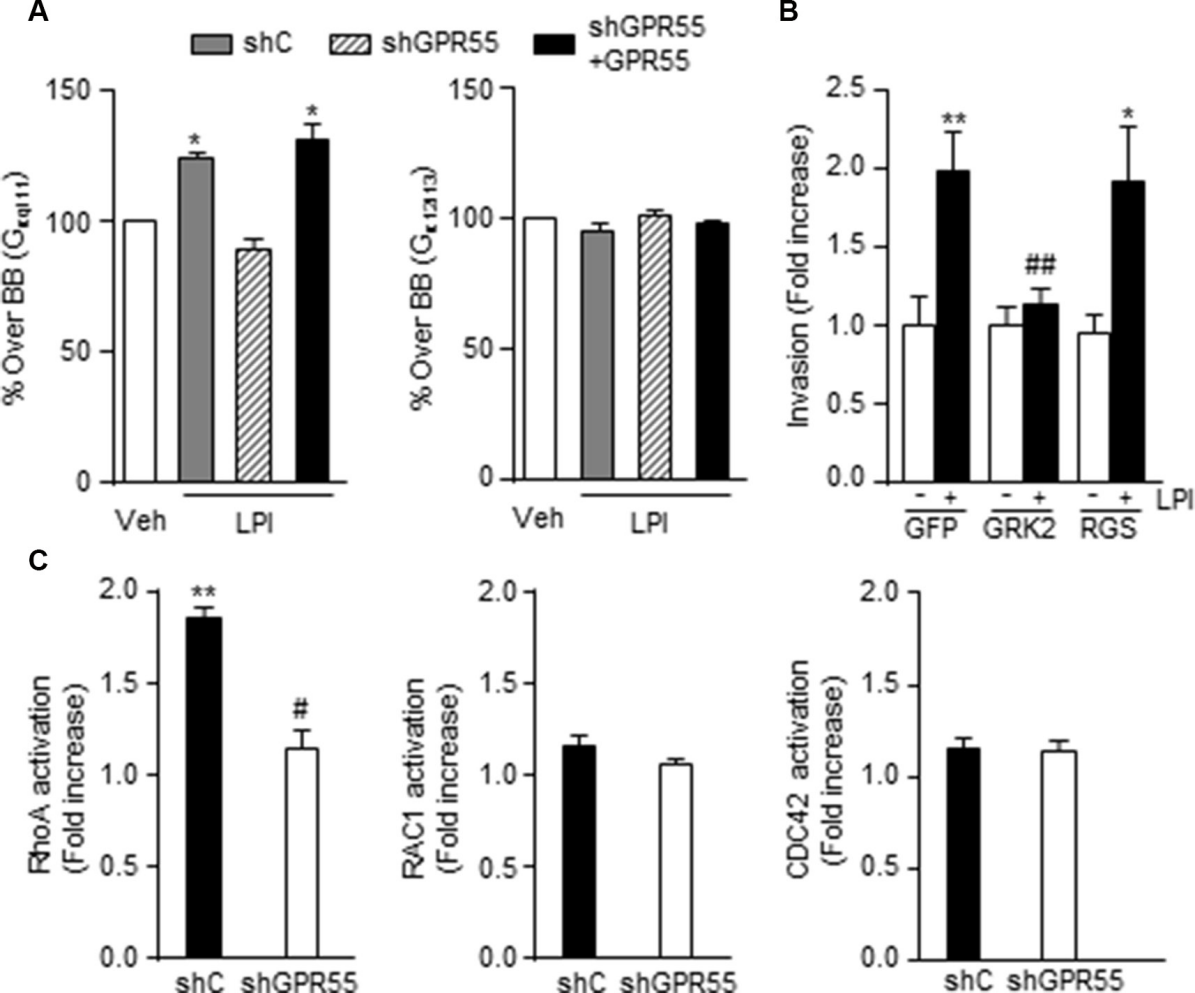
GPR55-driven pro-invasive responses involve coupling to G_q/11_ and activation of RhoA (**A**) Activation of G_q/11_ or G_12/13_ proteins by LPI (10^−5^ M) as determined by Antibody-capture [^35^S]GTPγS scintillation proximity assay (SPA). Results are expressed as percentage of [^35^S]GTPγS basal binding (BB, binding obtained in the absence of the agonist, set as 100% for each cell line, and represented as a single bar in the graphs) to the indicated subunit. (**B**) Invasion of shGPR55 + GPR55 cells after incubation with 0.5 μM LPI or the corresponding vehicle (PBS) for 24 h, in the presence of a construct blocking G_q/11_ (GRK2) or G_12/13_ signaling (RGS), or the corresponding empty vector (GFP). (**C**) Activation of the indicated small GTPases after a 3 min incubation with 0.5 μM LPI. Results are expressed as fold increase activation over the corresponding vehicle-treated cells, set at 1. *p < 0.05; **p < 0.01 *vs.* vehicle-treated cells. ##*p* < 0.01 *vs.* GFP LPI-treated cells. #*p* < 0.01 *vs.* LPI-treated cells.

The molecular mechanisms responsible for GPR55-driven pro-metastatic properties were then studied. First, we analyzed which heterotrimeric G protein GPR55 activates to mediate its actions. Previous reports had shown that this receptor couples to G_12/13_ [24–29] and G_q/11_ proteins [28] as its other closely related lysophospholipid receptors [30]. We therefore focused on these two families of G proteins, which are in fact intimately related to the regulation of cytoskeleton rearrangement, cell migration and invasion, and thus the generation of metastases [3–5]. By an antibody-capture [^35^S]GTPγS scintillation proximity assay (SPA) [31], we observed that LPI induced the coupling of GPR55 to G_q/11_ in MDA-MB-231 cells, an effect that was not observed in cells with reduced expression of the receptor and that was rescued in GPR55 overexpressing cells (Figure 3A, left). No coupling to G_12/13_ in response to LPI was observed in any of these cell lines (Figure 3A, right). The specific activation of G_q/11_ by GPR55 was further confirmed by the use of different chimeric constructs that behave as dominant-negative mutants for either G_q/11_ or G_12/13_. In this case, the increased invasiveness evoked by LPI in GPR55-overexpressing cells was prevented when G_q/11_ signaling was blocked by using a GFP-GRK2 chimeric construct (Figure 3B). In contrast, blockade of G_12/13_ signaling by means of a GFP-RGS chimera did not alter LPI-induced GPR55-mediated cancer cell invasion (Figure 3B). One of the main targets of G_q/11_ involved in actin remodeling, cell dynamics and metastasis is the Rho family of small GTPases, which consists of three subfamilies (Rho, Rac, and Cdc42) [5, 32]. GPR55 has been shown to activate Rho GTPases in non-cancer contexts [24, 26, 28, 29, 33, 34]. In our experimental setting, LPI significantly stimulated RhoA activity, and this effect was prevented by GPR55 knock-down (Figure 3C, left). In contrast, neither Rac1 (Figure 3C, middle) nor Cdc42 (Figure 3C, right) changed their activity upon LPI challenge (Figure 3C). Together, these findings support that GPR55 activation elicits pro-invasive responses in cancer cells by binding to G_q/11_ heterotrimeric G proteins and activating RhoA.

### GPR55 activates the transcription factor ETV4/PEA3 through coupling to G_q/11_ and stimulation of ERK

**Figure 4:**
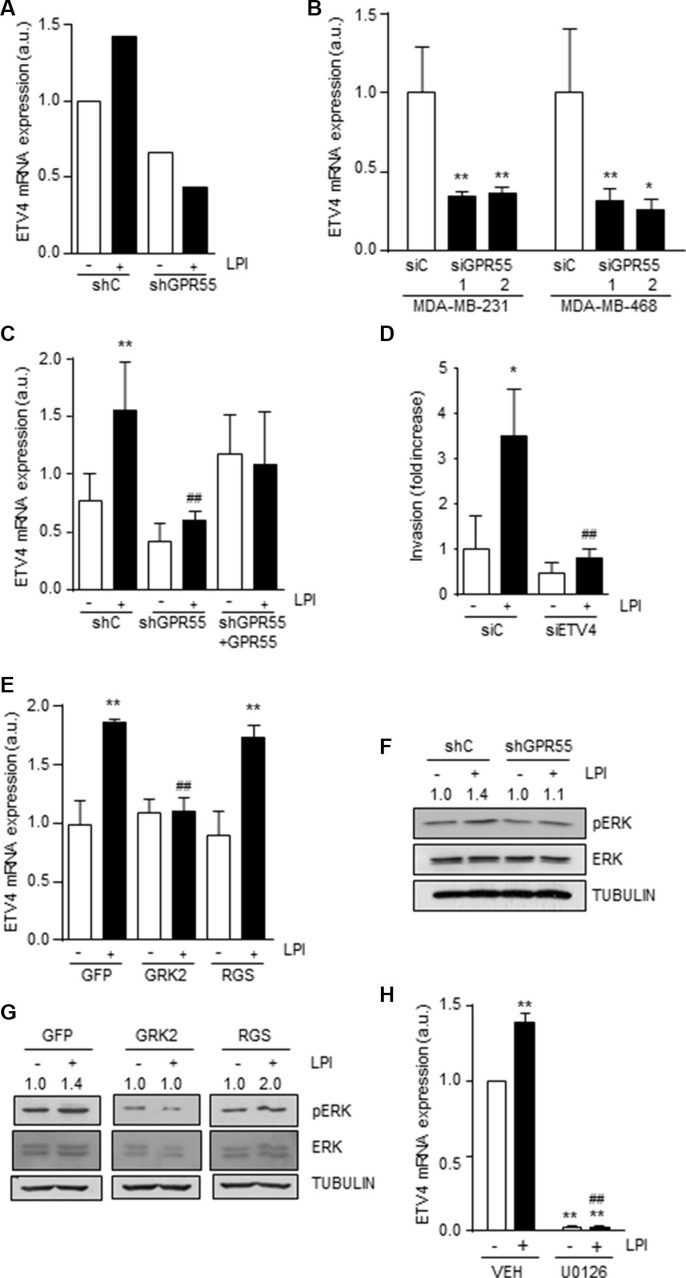
GPR55 activates the transcription factor ETV4/PEA3 through coupling to G_q/11_ and stimulation of ERK Relative ETV4 mRNA expression in the indicated cell lines (**A** and **B**), in the metastases derived from those cell lines (**C**) (see Figure 2G–2H for details on metastasis generation and treatment), and in shC cells transfected with constructs blocking blocking G_q/11_ (GRK2) or G_12/13_ signaling (RGS), or the corresponding empty vector (GFP) (E). In A, ETV4 expression was determined by the RT2 Profiler PCR Array of Human Tumor Metastases (see Supplementary Table S1 legend), and in (B), (**C** and **E**) by real-time quantitative PCR. In (B), siGPR55 1 and 2 represent two different GPR55 siRNAs. (D) Invasion of the indicated cell lines upon transient ETV4 knockdown. (**F** and **G**) Western blot analysis of phospho-ERK in shC and shGPR55 cells after treatment with 0.5 μM LPI or the corresponding vehicle (PBS) for 3 min (F) and shC cells after incubation with LPI and expressing the indicated G protein signaling blocking constructs (G). Representative luminograms are shown. Numbers on top of the images correspond to the densitometric analysis of pERK levels and are expressed as fold increase *vs.* the corresponding vehicle-treated cells, set at 1 (*n* = 3). (**H**) Relative ETV4 mRNA expression, as determined by real-time quantitative PCR, in shC cells challenged with 0.5 μM LPI and the MEK inhibitor U0126 (1μM) for 3 min. **p < 0.01 *vs.* siC or shC vehicle-treated cells. ##*p* < 0.01 *vs.* siC or shC LPI-treated cells (C), GFP LPI-treated cells (E) or LPI-treated cells (H).

To further characterize the downstream molecular players responsible for GPR55-driven pro-metastatic features, we analyzed the expression of a series of metastasis-related genes in response to LPI. By using a Human Metastasis PCR Array we found a group of genes that were modulated by LPI in GPR55-expressing cells (Supplementary Table S1). Among them, we focused our interest on those whose increased expression was ablated when GPR55 was silenced. One of them was the transcription factor ETV4/PEA3 (Figure 4A), which has been associated with metastasis in different tumor types [35, 36], including breast adenocarcinomas in general [37] and triple-negative breast cancers in particular [38]. First, we validated the reduction in ETV4 mRNA found after GPR55 stable knockdown by silencing the receptor transiently in MDA-MB-231 cells and an additional triple-negative breast cancer cell line (MDA-MB-468, Figure 4B), which ruled out the possibility that reduced ETV4 mRNA levels were a consequence of adaptive mechanisms to the chronic lack of GPR55 or a cell line-specific event. ETV4 mRNA expression was enhanced by LPI both in cell cultures (Figure 4A) and in the lung metastases generated in immunodeficient mice (Figure 4C), an effect that was not observed upon GPR55 knock-down (Figures 4A and 4C). Moreover, the increase in cell invasion produced by LPI was abolished by ETV4 transient knockdown (Figure 4D). In line with our previous data pointing to the involvement of G_q/11_ in LPI action, the increase in ETV4 levels produced by this GPR55 ligand was abrogated when G_q/11_ signaling was blocked with the GFP-GRK2 chimera (Figure 4E). It has been described that ETV4 expression is controlled by ERK in esophageal cancer [39] and so we found in our triple-negative breast cancer setting. Thus, LPI increased ERK phosphorylation (i.e. activation) via GPR55 (as demonstrated by the lack of such effect in GPR55 silenced cells; Figure 4F), and through the coupling to G_q/11_ (as demonstrated by the blockade of this action by the GFP-GRK2 construct; Figure 4G). Importantly, the increase in ETV4 mRNA levels induced by LPI was prevented by the inhibition of the ERK cascade with the MEK inhibitor U0126 (Figure 4H). Together, these results support that the prometastatic responses induced by GPR55 are mediated by the coupling to G_q/11_ and the subsequent activation of ERK, which in turn controls ETV4 expression.

## DISCUSSION

Our results show that activation of GPR55 promotes metastasis in breast cancer, thus supporting that this receptor may be exploited as a new target in oncology, specifically in the metastatic disease associated to the highly aggressive triple-negative breast cancer. Indirect links between GPR55 and metastasis have been previously suggested in other cancer types. For example, Piñeiro *et al.* showed that anchorage-independent growth (a crucial feature of cancer cells during metastatic spreading) of a prostate cancer cell line was inhibited by GPR55 downregulation [11]. More recently, Kargl *et al.* have reported that pharmacological and genetic blockade of GPR55 impairs migration of a colon cancer cell line *in vitro* and in an animal model of liver metastasis [17]. Regarding breast cancer specifically, Ford *et al.* demonstrated that LPI induces migration and elongation of MDA-MB-231 cells [16]. However, in the latter study it remained unclear whether the LPI effects were mediated by GPR55. Ford *et al.* also observed that the poorly metastatic MCF-7 cell line (which expresses low levels of GPR55) increases its migration capacity upon GPR55 overexpression, an effect that was increased by LPI and completely blocked by selective GPR55 knockdown [16]. The work presented here cogently shows that activation of GPR55 promotes metastatic responses *in vitro* and *in vivo* in triple-negative breast cancer. These results suggest that blockade of this receptor may be a useful strategy for the management of the metastatic disease in this population. The pharmacology of GPR55 is still quite controversial and most compounds with activity on this receptor bind to additional targets. It would be therefore desirable to find/synthesize compounds with blocking activity selectively on GPR55 to test their actual therapeutic potential.

Our findings also show that GPR55 may have not only predictive but prognostic value. Thus, an elevated GPR55 expression is associated with the basal/triple-negative breast cancer subtype, higher probability to develop metastases, and therefore poor patient prognosis. This notion that the LPI/GPR55 axis may represent a new marker with prognostic value is supported by additional observations. Regarding GPR55 itself, our group has previously observed that this receptor is upregulated in human skin tumors and other human squamous cell carcinomas compared with the corresponding healthy tissues [19], and that high GPR55 expression correlates with high histological grade in breast, pancreas and brain tumors, as well as with decreased overall survival in glioblastoma patients [10]. Additionally, increased levels of the proposed endogenous GPR55 ligand LPI [40], have been found in plasma and/or ascites from patients with colon [17] or ovarian cancer [41, 42], when compared with healthy subjects, or with women with non-cancerous pathologies. It would be very interesting to conduct similar studies in larger cohorts of breast cancer patients, which should include samples from the different molecular subtypes, to determine whether the association between GPR55 expression and poor prognosis in triple-negative cases that we report here occurs concomitant with elevated LPI plasma levels.

## MATERIALS AND METHODS

### Ethics statement

Investigation has been conducted in accordance with the ethical standards and according to the Declaration of Helsinki, and according to national and international guidelines, and has been approved by the authors' institutional review board.

### Tissue microarrays (TMAs)

PFA-fixed and paraffin-embedded blocks of tumor tissue from cases operated in the University Hospitals of Kiel between 1997 and 2010 were used for TMA construction. All patients gave informed consent, and the study was authorized by the Hospital's Ethics Committee. TMAs were generated by punching two 1 mm spots of each patient's sample. This resulted in a series of 483 tumor samples. Complete histopathological information was available for all the patients, including date and cause of death as well as date of local and/or distant relapse.

### Immunohistological analysis

Tissue sections were subjected to a heat-induced antigen retrieval step prior to exposure to an anti-GPR55 antibody (Abcam, Cambridge, UK). Immunodetection was performed using the Envision method with DAB as the chromogen. For GPR55 expression, cases were scored as 0 (no staining), 1 (weak staining), 2 (moderate staining), or 3 (high staining). Anti-GPR55 antibody specificity controls have been previously reported [12].

### Real-time quantitative PCR, and analysis of published microarray datasets

RNA was isolated with Trizol Reagent (Invitrogen, Barcelona, Spain) with the Real Star Kit (Durviz, Valencia, Spain), and cDNA was obtained with Transcriptor Reverse Transcriptase (Roche Applied Science, Barcelona, Spain). The primers used for GPR55 amplification were: sense 5′-CATGTGTTTCTCCAACGTCAA-3′ and antisense 5′-TGCGGAATTCTTTGATGACA-3′; and for ETV4 amplification: sense 5′-GCAGTTTGTTCCTGATTTCCA-3′ and antisense 5′-ACTCTGGGGCTCCTTCTTG-3′. Probes were from the Universal Probe Library (Roche Applied Science), and TATA Binding Protein (TBP) was used as reference: sense 5′-CCCATGACTCCCATGACC-3′ and antisense 5′-TTTACAACCAAGATTCACTGTGG-3′).

Human GPR55 mRNA expression was obtained from the microarray dataset published by the TCGA network in [20], downloaded from the cBio Cancer Genomics Portal [43]; from the microarray data set published in [21], downloaded from the Stanford Microarray Database (http://microarray-pubs.stanford.edu/wound_NKI/explore.html); and from database in [22], obtained through the European Genome-Phenome Archive (http://www.ebi.ac.uk/ega/) (accession number EGAS00000000083).

### Cell cultures

All the human breast adenocarcinoma cell lines were from ATCC-LGC (Barcelona, Spain). To stably knockdown GPR55 in MDA-MB-231 cells, a target-specific shRNA construct was used (GeneCopoeia, Rockville, MD). A scrambled shRNA construct was used as control (GeneCopoeia). Stably transfected cells were selected with puromycin. Transient GPR55 and ETV4 knockdown were carried out by using different ON-TARGETplus siRNAs (Dharmacon, Lafayette, CO): GPR55 siRNA1: 5′-GAAUUCCGCAUGAACAUCA-3′; GPR55 siRNA2: 5′-GAGAAACAGCUUUAUCGUA- 3′; for ETV4 a SMARTpool consisting of 4 different siRNAs was used: 5′-GGGCAGAGCAACGGAAUUU-3′, 5′-GAAU GGAGUUCAAGCUCAU-3′, 5′-GGACUUCGCCUAC GACUCA-3′ and 5′-GAUGAAAGCCGGAUACUUG- 3′. A non-targeted siRNA was used as control: 5′-UUCU CCGAACGUGUCACGUtt-3′. For the generation of MDA-MB-231 cells overexpressing GPR55, a 3xHA-GPR55 plasmid, or the corresponding empty vector (pcDNA3, Invitrogen) were used. All transfections were performed with Lipofectamine 2000 (Invitrogen). The graphs showing the resulting GPR55 or ETV4 mRNA levels are shown in Supplementary Figure S1. All cell lines were maintained in DMEM supplemented with 10% FBS.

The chimeric constructs to block G_q/11_ or G_12/13_ signaling were generated by fusion of a GFP containing pCEFL plasmid to the RGS domain of GRK2 (GFP-GRK2) or to the RGS domain of PDZ-RhoGFP (GFP-RGS) [44].

### Cell invasion

Cell invasion assays were performed in BD BioCoat^TM^ Matrigel^TM^ Invasion Chambers (BD Biosciences, Bedford, MA). Transwell inserts were placed onto 24-well, glass bottom greiner multiwell plates. Chemoattractants (10% FBS or 0.5 mM LPI) were added into the wells, and serum starved cells were placed into the inserts. After 24 h incubation, cells were fixed in 4% formaldehyde and stained with DAPI (Roche Pharma, Madrid, Spain). Images were taken with a confocal microscope (Leica TCS SP5) using a 20X DRY 0.7 NA objective. Capturing conditions were set up in two independent channels for DAPI stained cells and insert membrane reflection. Four equidistant non-overlapping fields were acquired close to the center of each insert avoiding possible influences from borders. For each field a volume big enough to contain all (migrating and non-migrating) cells was captured with a Z distance between images of 1 micron (small enough relative to nuclei size to make sure no nuclei was left unnoticed). All stack fields were visualized using Imaris x64 7.3.1 software. The center of every cell was recorded relative to the transwell insert membrane and cells were classified as either migrating or non migrating cells. Cells in touch with image borders were discarded.

### Chemotaxis assays

2D chemotaxis μ-slide chambers (Ibidi, Munich, Germany) were precoated with collagen and cells where allowed to adhere and serum starve overnight. Incomplete media alone or supplemented with LPI (1 μM) were located in the reservoir chambers. Cell migration was imaged by time lapse microscopy (IX83 inverted microscope, Olympus; 20x magnification) at 5 minutes intervals over a period of up to 20 hours. Image J Manual Tracking plug-in and Chemotaxis Migration tool (Ibidi) were used to quantify the cell trajectories.

### Generation of lung metastases

Lung metastases were generated by injection of the different luciferase expressing cell lines (5 × 10^5^) into the lateral tail vein of 6 week-old NOD/SCID female mice. Starting from day 2 after cell injection, half of the animals of each experimental group received LPI treatment (12 μg, i.p., three times per week) and the other half treatment with the corresponding vehicle (PBS). Forty five days after cell injection, animals were analyzed by bioluminescence after an intraperitoneal injection of D-Luciferin (Sigma) in an IVIS 2000 system (Xenogen Corp, Alameda, CA). Imaging data were processed with Living Image software (Xenogen Corp).

### Antibody-capture [^35^S]GTPγS scintillation proximity assay (SPA)

SPAs were performed as previously described [45]. Briefly, cell membrane homogenates were incubated in 96-well Isoplates (Perkin Elmer Life Sciences, Maanstraat, Germany) in incubation buffer containing 0.4 nM [^35^S] GTP_γ_S (Perkin Elmer) and 50 or 100 μM GDP for G_q/11_ or G_12/13_ proteins, respectively. Specific antibodies for each G_α_ subunit (rabbit polyclonal anti-G_αq/11_ and rabbit polyclonal anti-G_α12/13_; Santa Cruz Biotechnologies, Madrid, Spain) and PVT SPA beads coated with protein A (Perkin Elmer) were used. Radioactivity was quantified on a MicroBeta TriLux scintillation counter (Perkin Elmer).

### Activity of Rho family GTPases

The activity of the Rho family of small GTPases was determined by RhoA/Rac1/Cdc42 Activation Assay Combo Biochem Kit™ (Cytoskeleton, Denver, CO) following manufacturer's instructions.

### Human metastasis PCR array

The expression of metastasis related genes after treatment with LPI (0, 5 μM, 20 h) or the corresponding vehicle (PBS) was determined by the RT2 Profiler PCR Array of Human Tumor Metastases (ref. 524 PAHS-028Z-4, Qiagen, Valencia, CA). RNA from cell cultures was isolated with Trizol reagent (Sigma, St. Louis, MO), including a DNA digestion step with genomic DNA elimination mix (Qiagen). cDNA was subsequently obtained with a RT2 first strand kit according to manufacturer's instructions (Qiagen). Real-time PCR assay was performed using the RT2 profiler PCR array in combination with RT2 SYBR Green master mix (Qiagen). Amplifications were run in a 7900 HT-fast real-time PCR system (Applied Biosystems, Carlsbard, CA), and data were analyzed using the SABiosciences PCR array data analysis template Excel (Qiagen).

### Western blot analysis

Lysates from the different cell lines or lungs were obtained, and proteins were separated by SDS–PAGE and then transferred onto PVDF membranes. Blots were incubated with anti-phospho-ERK (Thr202/Tyr204), anti-ERK (Cell Signaling Technology, Danvers, MA, USA) and anti-α-Tubulin (TUB) (used as loading control, Sigma-Aldrich). Luminograms were obtained with the Amersham Enhanced Chemiluminescence Detection Kit (GE Healthcare, Uppsala, Sweden), and densitometric analysis was performed with Quantity One software (Bio-Rad, Madrid, Spain).

### Statistical analysis

The Pearson's chi-squared test was used for the analysis of the association between GPR55 expression and the molecular features of the breast tumor samples included in the TMA. Kaplan-Meier survival curves were statistically compared by the log-rank test. Analysis of variance (ANOVA) with a post hoc analysis by the Student-Newman-Keuls' test was routinely used for the rest of the analyses.

## SUPPLEMENTARY MATERIALS



## References

[1] Alexander SP, Benson HE, Faccenda E, Pawson AJ, Sharman JL, Spedding M, Peters JA, Harmar AJ (2013). The Concise Guide to PHARMACOLOGY 2013/14: G protein-coupled receptors. Br J Pharmacol.

[2] Rask-Andersen M, Masuram S, Schioth HB (2014). The druggable genome: Evaluation of drug targets in clinical trials suggests major shifts in molecular class and indication. Annu Rev Pharmacol Toxicol.

[3] Dorsam RT, Gutkind JS (2007). G-protein-coupled receptors and cancer. Nat Rev Cancer.

[4] Lappano R, Maggiolini M (2011). G protein-coupled receptors: novel targets for drug discovery in cancer. Nat Rev Drug Discov.

[5] O'Hayre M, Degese MS, Gutkind JS (2014). Novel insights into G protein and G protein-coupled receptor signaling in cancer. Curr Opin Cell Biol.

[6] Balenga NA, Henstridge CM, Kargl J, Waldhoer M (2011). Pharmacology signaling and physiological relevance of the G protein-coupled receptor 55. Adv Pharmacol.

[7] Henstridge CM, Balenga NA, Kargl J, Andradas C, Brown AJ, Irving A, Sanchez C, Waldhoer M (2011). Minireview: recent developments in the physiology and pathology of the lysophosphatidylinositol-sensitive receptor GPR55. Mol Endocrinol.

[8] Pertwee RG, Howlett AC, Abood ME, Alexander SP, Di Marzo V, Elphick MR, Greasley PJ, Hansen HS, Kunos G, Mackie K, Mechoulam R, Ross RA (2010). International Union of Basic and Clinical Pharmacology LXXIX Cannabinoid receptors and their ligands: beyond CB and CB. Pharmacol Rev.

[9] Alexander SP, Davenport AP, Kelly E, Marrion N, Peters JA, Benson HE, Faccenda E, Pawson AJ, Sharman JL, Southan C, Davies JA, Collaborators C (2015). The Concise Guide to PHARMACOLOGY 2015/16: G protein-coupled receptors. Br J Pharmacol.

[10] Andradas C, Caffarel MM, Perez-Gomez E, Salazar M, Lorente M, Velasco G, Guzman M, Sanchez C (2011). The orphan G protein-coupled receptor GPR55 promotes cancer cell proliferation via ERK. Oncogene.

[11] Pineiro R, Maffucci T, Falasca M (2011). The putative cannabinoid receptor GPR55 defines a novel autocrine loop in cancer cell proliferation. Oncogene.

[12] Perez-Gomez E, Andradas C, Flores JM, Quintanilla M, Paramio JM, Guzman M, Sanchez C (2013). The orphan receptor GPR55 drives skin carcinogenesis and is upregulated in human squamous cell carcinomas. Oncogene.

[13] Adinolfi B, Romanini A, Vanni A, Martinotti E, Chicca A, Fogli S, Nieri P (2013). Anticancer activity of anandamide in human cutaneous melanoma cells. Eur J Pharmacol.

[14] He D, Wang J, Zhang C, Shan B, Deng X, Li B, Zhou Y, Chen W, Hong J, Gao Y, Chen Z, Duan C (2015). Down-regulation of miR-675–5p contributes to tumor progression and development by targeting pro-tumorigenic GPR55 in non-small cell lung cancer. Mol Cancer.

[15] Hofmann NA, Yang J, Trauger SA, Nakayama H, Huang L, Strunk D, Moses MA, Klagsbrun M, Bischoff J, Graier WF (2015). The GPR 55 agonist L-alpha-lysophosphatidylinositol mediates ovarian carcinoma cell-induced angiogenesis. Br J Pharmacol.

[16] Ford LA, Roelofs AJ, Anavi-Goffer S, Mowat L, Simpson DG, Irving AJ, Rogers MJ, Rajnicek AM, Ross RA (2010). A role for L-alpha-lysophosphatidylinositol and GPR55 in the modulation of migration orientation and polarization of human breast cancer cells. Br J Pharmacol.

[17] Kargl J, Andersen L, Hasenohrl C, Feuersinger D, Stancic A, Fauland A, Magnes C, El-Heliebi A, Lax S, Uranitsch S, Haybaeck J, Heinemann A, Schicho R (2016). GPR55 promotes migration and adhesion of colon cancer cells indicating a role in metastasis. Br J Pharmacol.

[18] International Agency for Research on Cancer (IARC) and World Health Organization (WHO) GLOBOCAN 2012: Estimated cancer incidence mortality and prevalence worldwide in 2012.

[19] Perez-Gomez E, Andradas C, Blasco-Benito S, Caffarel MM, Garcia-Taboada E, Villa-Morales M, Moreno E, Hamann S, Martin-Villar E, Flores JM, Wenners A, Alkatout I, Klapper W (2015). Role of cannabinoid receptor CB2 in HER2 pro-oncogenic signaling in breast cancer. J Natl Cancer Inst.

[20] Network TCGA (2012). Comprehensive molecular portraits of human breast tumours. Nature.

[21] van de Vijver MJ, He YD, van't Veer LJ, Dai H, Hart AA, Voskuil DW, Schreiber GJ, Peterse JL, Roberts C, Marton MJ, Parrish M, Atsma D, Witteveen A (2002). A gene-expression signature as a predictor of survival in breast cancer. N Engl J Med.

[22] Curtis C, Shah SP, Chin SF, Turashvili G, Rueda OM, Dunning MJ, Speed D, Lynch AG, Samarajiwa S, Yuan Y, Graf S, Ha G, Haffari G (2012). The genomic and transcriptomic architecture of 2000 breast tumours reveals novel subgroups. Nature.

[23] Hanahan D, Weinberg RA (2000). The hallmarks of cancer. Cell.

[24] Balenga NA, Aflaki E, Kargl J, Platzer W, Schroder R, Blattermann S, Kostenis E, Brown AJ, Heinemann A, Waldhoer M (2011). GPR55 regulates cannabinoid 2 receptor-mediated responses in human neutrophils. Cell Res.

[25] Brown AJ, Daniels DA, Kassim M, Brown S, Haslam CP, Terrell VR, Brown J, Nichols PL, Staton PC, Wise A, Dowell SJ (2011). Pharmacology of GPR55 in yeast and identification of GSK494581A as a mixed-activity glycine transporter subtype 1 inhibitor and GPR55 agonist. J Pharmacol Exp Ther.

[26] Henstridge CM, Balenga NA, Ford LA, Ross RA, Waldhoer M, Irving AJ (2009). The GPR55 ligand L-alpha-lysophosphatidylinositol promotes RhoA-dependent Ca2+ signaling and NFAT activation. FASEB J.

[27] Huang L, Ramirez JC, Frampton GA, Golden LE, Quinn MA, Pae HY, Horvat D, Liang LJ, Demorrow S (2011). Anandamide exerts its antiproliferative actions on cholangiocarcinoma by activation of the GPR55 receptor. Lab Invest.

[28] Lauckner JE, Jensen JB, Chen HY, Lu HC, Hille B, Mackie K (2008). GPR55 is a cannabinoid receptor that increases intracellular calcium and inhibits M current. Proc Natl Acad Sci U S A.

[29] Ryberg E, Larsson N, Sjogren S, Hjorth S, Hermansson NO, Leonova J, Elebring T, Nilsson K, Drmota T, Greasley PJ (2007). The orphan receptor GPR55 is a novel cannabinoid receptor. Br J Pharmacol.

[30] Choi JW, Herr DR, Noguchi K, Yung YC, Lee CW, Mutoh T, Lin ME, Teo ST, Park KE, Mosley AN, Chun J (2010). LPA receptors: subtypes and biological actions. Annu Rev Pharmacol Toxicol.

[31] DeLapp NW, McKinzie JH, Sawyer BD, Vandergriff A, Falcone J, McClure D, Felder CC (1999). Determination of [35S] guanosine-5′-O-(3-thio)triphosphate binding mediated by cholinergic muscarinic receptors in membranes from Chinese hamster ovary cells and rat striatum using an anti-G protein scintillation proximity assay. J Pharmacol Exp Ther.

[32] Vega FM, Ridley AJ (2008). Rho GTPases in cancer cell biology. FEBS Lett.

[33] Oka S, Kimura S, Toshida T, Ota R, Yamashita A, Sugiura T (2010). Lysophosphatidylinositol Induces Rapid Phosphorylation of p38 Mitogen-Activated Protein Kinase and Activating Transcription Factor 2 in HEK293 Cells Expressing GPR55 and IM-9 Lymphoblastoid Cells. J Biochem.

[34] Whyte LS, Ryberg E, Sims NA, Ridge SA, Mackie K, Greasley PJ, Ross RA, Rogers MJ (2009). The putative cannabinoid receptor GPR55 affects osteoclast function *in vitro* and bone mass *in vivo*. Proc Natl Acad Sci U S A.

[35] de Launoit Y, Baert JL, Chotteau-Lelievre A, Monte D, Coutte L, Mauen S, Firlej V, Degerny C, Verreman K (2006). The Ets transcription factors of the PEA3 group: transcriptional regulators in metastasis. Biochim Biophys Acta.

[36] Shindoh M, Higashino F, Kohgo T (2004). E1AF an ets-oncogene family transcription factor. Cancer Lett.

[37] Yuen HF, Chan YK, Grills C, McCrudden CM, Gunasekharan V, Shi Z, Wong AS, Lappin TR, Chan KW, Fennell DA, Khoo US, Johnston PG, El-Tanani M (2011). Polyomavirus enhancer activator 3 protein promotes breast cancer metastatic progression through Snail-induced epithelial-mesenchymal transition. J Pathol.

[38] Yuan ZY, Dai T, Wang SS, Peng RJ, Li XH, Qin T, Song LB, Wang X (2014). Overexpression of ETV4 protein in triple-negative breast cancer is associated with a higher risk of distant metastasis. Onco Targets Ther.

[39] Keld R, Guo B, Downey P, Gulmann C, Ang YS, Sharrocks AD (2010). The ERK MAP kinase-PEA3/ETV4-MMP-1 axis is operative in oesophageal adenocarcinoma. Mol Cancer.

[40] Oka S, Nakajima K, Yamashita A, Kishimoto S, Sugiura T (2007). Identification of GPR55 as a lysophosphatidylinositol receptor. Biochem Biophys Res Commun.

[41] Sutphen R, Xu Y, Wilbanks GD, Fiorica J, Grendys EC, LaPolla JP, Arango H, Hoffman MS, Martino M, Wakeley K, Griffin D, Blanco RW (2004). Lysophospholipids are potential biomarkers of ovarian cancer. Cancer Epidemiol Biomarkers Prev.

[42] Xiao YJ, Schwartz B, Washington M, Kennedy A, Webster K, Belinson J, Xu Y (2001). Electrospray ionization mass spectrometry analysis of lysophospholipids in human ascitic fluids: comparison of the lysophospholipid contents in malignant vs nonmalignant ascitic fluids. Anal Biochem.

[43] Cerami E, Gao J, Dogrusoz U, Gross BE, Sumer SO, Aksoy BA, Jacobsen A, Byrne CJ, Heuer ML, Larsson E, Antipin Y, Reva B, Goldberg AP (2012). The cBio cancer genomics portal: an open platform for exploring multidimensional cancer genomics data. Cancer Discov.

[44] Yagi H, Tan W, Dillenburg-Pilla P, Armando S, Amornphimoltham P, Simaan M, Weigert R, Molinolo AA, Bouvier M, Gutkind JS (2011). A synthetic biology approach reveals a CXCR4-G13-Rho signaling axis driving transendothelial migration of metastatic breast cancer cells. Sci Signal.

[45] Erdozain AM, Diez-Alarcia R, Meana JJ, Callado LF (2012). The inverse agonist effect of rimonabant on G protein activation is not mediated by the cannabinoid CB1 receptor: evidence from postmortem human brain. Biochem Pharmacol.

