# Supplementary material for: Activation of the orphan receptor GPR55 by lysophosphatidylinositol promotes metastasis in triple-negative breast cancer
Source: Oncotarget. 2016 Jun 21;7(30):47565–75. doi: 10.18632/oncotarget.10206 (PMC5216961; doi:10.18632/oncotarget.10206)
Supplement: Supplementary file 1 [file oncotarget-07-47565-s001.pdf]

# Activation of the orphan receptor GPR55 by lysophosphatidylinositol promotes metastasis in triple-negative breast cancer

## Supplementary Materials

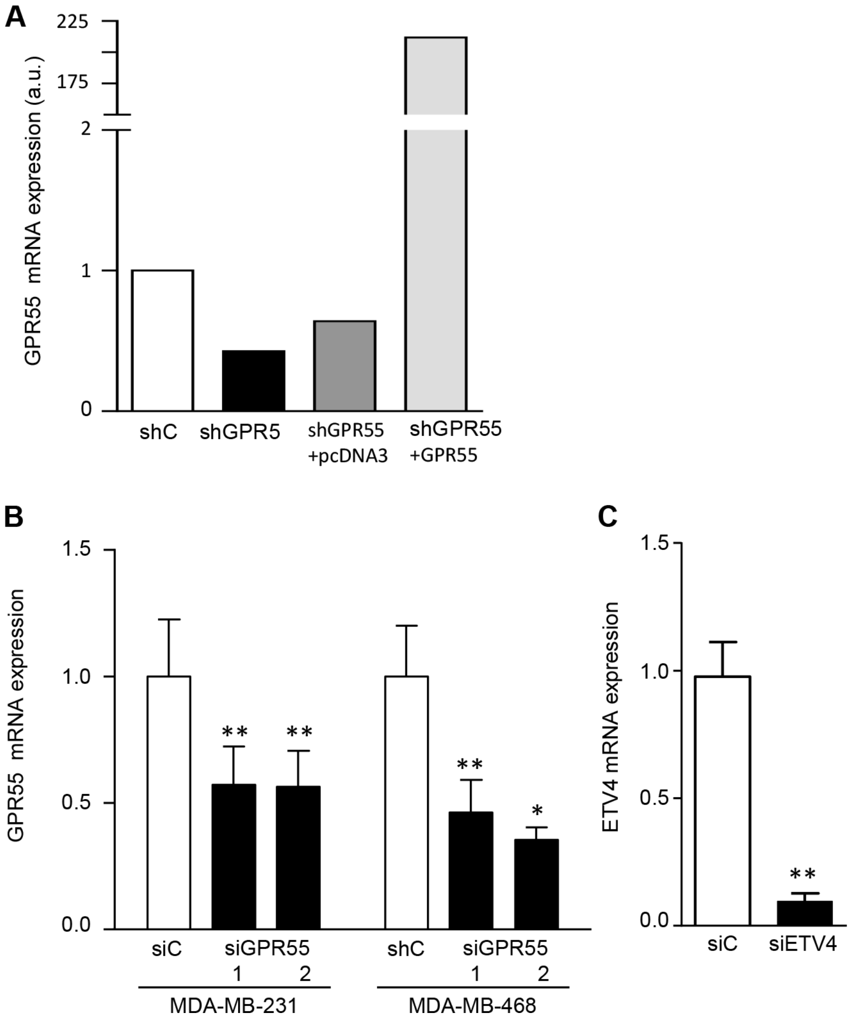


**Supplementary Figure S1: Real-time quantitative PCR analysis of GPR55 and ETV4 mRNA expression.** (**A**) Relative GPR55 expression in MDA-MB-231 cells stably knocked-down with a selective GPR55 shRNA (shGPR55), and co-transfected with a GPR55 overexpression plasmid (shGPR55+GPR55) or the corresponding empty vector (shGPR55+pcDNA3). shC represents cells transfected with a non-targeted shRNA. (**B**) Relative GPR55 expression in MDA-MB-231 or MDA-MB-468 cells transiently transfected with two different GPR55 selective siRNAs (1 and 2) or a non-targeted siRNA (siC). (**C**) Relative ETV4 expression in MDA-MB-231 cells after transient ETV4 knockdown with a selective siRNA (siETV4) or a non-targeted siRNA (siC). Results are expressed in arbitrary units vs shC or siC cells, set at 1. $^{*}p < 0.05$; $^{**}p < 0.01$ vs. siC cells.

**Supplementary Table S1: Expression of metastasis related genes in shC and shGPR55 cells after treatment with LPI (0, 5 μM, 20 h) or the corresponding vehicle (PBS)**

A

| Symbol | Up/Down fold regulation (vs shC veh) | | |
|---|---|---|---|
| | shC LPI | shGPR55 veh | shGPR55 LPI |
| APC | −1,2092 | −1,1896 | −1,1057 |
| BRMS1 | 1,1634 | 1,078 | 1,281 |
| CCL7 | 2,1723 | 6,1131 | −1,0756 |
| CD44 | 1,0184 | −1,1425 | 1,3741 |
| CD82 | 1,0247 | −1,2528 | −1,0272 |
| CDH1 | 2,1932 | −4,0037 | 2,8836 |
| CDH11 | −1,2087 | −2,1704 | −1,8447 |
| CDH6 | −1,2297 | 1,0945 | 1,9366 |
| CDKN2A | −1,2297 | 1,0945 | −1,0756 |
| CHD4 | −1,02 | −1,1934 | −1,4715 |
| COL4A2 | 1,0385 | 1,0668 | 1,1721 |
| CST7 | −1,533 | 1,3993 | 1,573 |
| CTBP1 | 1,1154 | −1,1847 | −1,0685 |
| CTNNA1 | 1,1028 | 1,0705 | −1,1054 |
| CTSK | −1,1459 | 1,2039 | 1,0713 |
| CTSL | −1,0132 | −1,0241 | 1,1174 |
| CXCL12 | −6,4502 | 1,2418 | −1,3388 |
| CXCR2 | −1,137 | −1,0025 | −1,0267 |
| CXCR4 | −1,1172 | −1,6578 | −1,2743 |
| DENR | −1,0227 | 1,0527 | 1,2738 |
| EPHB2 | 1,3194 | 1,0519 | 1,0273 |
| ETV4 | 1,4311 | −1,3384 | −1,5655 |
| EWSR1 | 1,0305 | −1,0997 | −1,0165 |
| FAT1 | 1,0067 | −1,1077 | −1,1355 |
| FGFR4 | 2,0421 | −1,8312 | −1,6706 |
| FLT4 | −1,2297 | 11,883 | 3,2939 |
| FN1 | 1,1433 | −1,1804 | −1,0867 |
| FXYD5 | 1,0897 | −1,0026 | 1,1596 |
| GNRH1 | 1,4179 | 1,4779 | 1,3964 |
| HGF | −1,2297 | 1,0945 | −1,0756 |
| HPSE | 1,1104 | −1,1166 | 1,0753 |
| HRAS | 1,1207 | 1,1725 | 1,2359 |
| HTATIP2 | −1,083 | 1,0201 | 1,1703 |
| IGF1 | 1,236 | 2,5581 | 1,6246 |
| IL18 | −1,0105 | 1,028 | 1,2475 |
| IL1B | 1,663 | 1,048 | −1,0134 |
| ITGA7 | −1,1785 | −3,2597 | −1,6463 |
| ITGB3 | 1,2609 | −1,2623 | 1,5225 |
| KISS1 | −1,0639 | −1,2337 | −1,0158 |
| KISS1R | −1,4096 | −1,0474 | 2,9119 |
| KRAS | 1,0667 | −1,0333 | 1,0603 |
| MCAM | 1,0475 | −1,1022 | −1,0188 |

B

| Symbol | Up/Down fold regulation | |
|---|---|---|
| | shC (LPI vs veh) | shGPR55 (LPI vs veh) |
| APC | −1,2092 | 1,0759 |
| BRMS1 | 1,1634 | 1,1883 |
| CCL7 | 2,1723 | −6,5752 |
| CD44 | 1,0184 | 1,5699 |
| CD82 | 1,0247 | 1,2196 |
| CDH1 | 2,1932 | 11,545 |
| CDH11 | −1,2087 | 1,1766 |
| CDH6 | −1,2297 | 1,7694 |
| CDKN2A | −1,2297 | −1,1772 |
| CHD4 | −1,02 | −1,2331 |
| COL4A2 | 1,0385 | 1,0987 |
| CST7 | −1,533 | 1,1241 |
| CTBP1 | 1,1154 | 1,1088 |
| CTNNA1 | 1,1028 | −1,1833 |
| CTSK | −1,1459 | −1,1237 |
| CTSL | −1,0132 | 1,1444 |
| CXCL12 | −6,4502 | −1,6625 |
| CXCR2 | −1,137 | −1,0241 |
| CXCR4 | −1,1172 | 1,301 |
| DENR | −1,0227 | 1,2101 |
| EPHB2 | 1,3194 | −1,0239 |
| ETV4 | 1,4311 | −1,1697 |
| EWSR1 | 1,0305 | 1,0818 |
| FAT1 | 1,0067 | −1,0251 |
| FGFR4 | 2,0421 | 1,0961 |
| FLT4 | −1,2297 | −3,6076 |
| FN1 | 1,1433 | 1,0862 |
| FXYD5 | 1,0897 | 1,1626 |
| GNRH1 | 1,4179 | −1,0584 |
| HGF | −1,2297 | −1,1772 |
| HPSE | 1,1104 | 1,2006 |
| HRAS | 1,1207 | 1,0541 |
| HTATIP2 | −1,083 | 1,1472 |
| IGF1 | 1,236 | −1,5746 |
| IL18 | −1,0105 | 1,2135 |
| IL1B | 1,663 | −1,062 |
| ITGA7 | −1,1785 | 1,9801 |
| ITGB3 | 1,2609 | 1,9219 |
| KISS1 | −1,0639 | 1,2146 |
| KISS1R | −1,4096 | 3,0499 |
| KRAS | 1,0667 | 1,0956 |
| MCAM | 1,0475 | 1,0818 |

| | | | |
|---|---|---|---|
| MDM2 | 1,1424 | −1,0518 | 1,1384 |
| MET | 1,1099 | 1,0028 | 1,2153 |
| METAP2 | 1,1328 | 1,0819 | 1,1358 |
| MGAT5 | 1,0932 | −1,0695 | −1,1429 |
| MMP10 | 1,7932 | 1,2597 | 1,6619 |
| MMP11 | 1,0349 | −1,0882 | −1,6464 |
| MMP13 | 1,1597 | −1,4543 | −1,7966 |
| MMP2 | 3,1206 | −1,238 | −2,9969 |
| MMP3 | 1,3103 | 1,2078 | 1,3631 |
| MMP7 | 1,7656 | 105,8178 | −1,0756 |
| MMP9 | 1,1601 | −3,8732 | −2,9399 |
| MTA1 | 1,1966 | −1,234 | 1,0103 |
| MTSS1 | 1,1124 | −1,0105 | 1,1732 |
| MYC | −1,0937 | 1,0742 | −1,075 |
| MYCL | −1,3414 | −3,1527 | −3,011 |
| NF2 | 1,2291 | 1,0023 | 1,1911 |
| NME1 | −1,0257 | 1,0602 | 1,1465 |
| NME4 | −1,0666 | 1,1023 | 1,1282 |
| NR4A3 | 1,3178 | 1,3068 | −1,9376 |
| PLAUR | −1,0104 | 1,0007 | −1,1369 |
| PNN | −1,0493 | −1,0856 | −1,132 |
| PTEN | 1,0332 | −1,1503 | 1,1698 |
| RB1 | 1,0648 | −1,0027 | 1,0437 |
| RORB | 1,1365 | −1,0836 | 1,1657 |
| RPSA | −1,0299 | −1,0619 | 1,093 |
| SERPINE1 | −1,1261 | −1,1879 | −1,4581 |
| SET | −1,047 | −1,0764 | −1,0638 |
| SMAD2 | −1,0114 | −1,224 | −1,3462 |
| SMAD4 | 1,0724 | −1,1516 | −1,16 |
| SRC | 1,2991 | −1,2366 | −1,2173 |
| SSTR2 | 1,5917 | 1,2097 | 1,1131 |
| SYK | −1,2297 | 1,0945 | −1,0756 |
| TCF20 | 1,1398 | 1,1414 | 1,3244 |
| TGFB1 | 1,1289 | −1,1359 | −1,1007 |
| TIMP2 | −1,0113 | 1,054 | 1,0911 |
| TIMP3 | −1,1525 | −1,247 | −1,1833 |
| TIMP4 | −1,167 | −1,6515 | −1,7873 |
| TNFSF10 | 4,6252 | 1,0945 | −1,0756 |
| TP53 | 1,088 | −1,1279 | −1,119 |
| TRPM1 | −1,2297 | 1,0945 | −1,0756 |
| TSHR | −8,8887 | −6,6045 | −2,6671 |
| VEGFA | 1,0427 | 1,17 | 1,2441 |
| ACTB | −1,0372 | −1,1045 | −1,3736 |
| B2M | −1,0827 | −1,0162 | 1,0164 |
| GAPDH | 1,0527 | −1,0816 | −1,0842 |
| HPRT1 | 1,0812 | 1,1139 | 1,2888 |
| RPLP0 | −1,0135 | 1,0899 | 1,1369 |

| | | |
|---|---|---|
| MDM2 | 1,1424 | 1,1974 |
| MET | 1,1099 | 1,2119 |
| METAP2 | 1,1328 | 1,0498 |
| MGAT5 | 1,0932 | −1,0687 |
| MMP10 | 1,7932 | 1,3193 |
| MMP11 | 1,0349 | −1,5129 |
| MMP13 | 1,1597 | −1,2354 |
| MMP2 | 3,1206 | −2,4207 |
| MMP3 | 1,3103 | 1,1286 |
| MMP7 | 1,7656 | −113,8161 |
| MMP9 | 1,1601 | 1,3175 |
| MTA1 | 1,1966 | 1,2467 |
| MTSS1 | 1,1124 | 1,1854 |
| MYC | −1,0937 | −1,1548 |
| MYCL | −1,3414 | 1,0471 |
| NF2 | 1,2291 | 1,1884 |
| NME1 | −1,0257 | 1,0814 |
| NME4 | −1,0666 | 1,0235 |
| NR4A3 | 1,3178 | −2,5322 |
| PLAUR | −1,0104 | −1,1377 |
| PNN | −1,0493 | −1,0428 |
| PTEN | 1,0332 | 1,3456 |
| RB1 | 1,0648 | 1,0465 |
| RORB | 1,1365 | 1,2632 |
| RPSA | −1,0299 | 1,1607 |
| SERPINE1 | −1,1261 | −1,2274 |
| SET | −1,047 | 1,0118 |
| SMAD2 | −1,0114 | −1,0999 |
| SMAD4 | 1,0724 | −1,0073 |
| SRC | 1,2991 | 1,0158 |
| SSTR2 | 1,5917 | −1,0868 |
| SYK | −1,2297 | −1,1772 |
| TCF20 | 1,1398 | 1,1603 |
| TGFB1 | 1,1289 | 1,0319 |
| TIMP2 | −1,0113 | 1,0352 |
| TIMP3 | −1,1525 | 1,0538 |
| TIMP4 | −1,167 | −1,0823 |
| TNFSF10 | 4,6252 | −1,1772 |
| TP53 | 1,088 | 1,0079 |
| TRPM1 | −1,2297 | −1,1772 |
| TSHR | −8,8887 | 2,4763 |
| VEGFA | 1,0427 | 1,0634 |
| ACTB | −1,0372 | −1,2436 |
| B2M | −1,0827 | 1,0329 |
| GAPDH | 1,0527 | −1,0023 |
| HPRT1 | 1,0812 | 1,157 |
| RPLP0 | −1,0135 | 1,0431 |

| | | | |
|---|---|---|---|
| HGDC | −1,2297 | 1,0945 | −1,0756 |
| RTC | 1,0004 | 1,1244 | −1,1569 |
| RTC | 1,0376 | 1,2187 | −1,2114 |
| RTC | 1,0451 | 1,2024 | −1,1069 |
| PPC | −1,2423 | 1,069 | 1,0239 |
| PPC | −1,1334 | 1,0323 | −1,0304 |
| PPC | −1,3639 | 1,0245 | −1,0451 |

| | | |
|---|---|---|
| HGDC | −1,2297 | −1,1772 |
| RTC | 1,0004 | −1,3009 |
| RTC | 1,0376 | −1,4764 |
| RTC | 1,0451 | −1,3309 |
| PPC | −1,2423 | −1,0441 |
| PPC | −1,1334 | −1,0637 |
| PPC | −1,3639 | −1,0707 |

Up/Down regulation is expressed as fold change in the expression of the indicated gene (up-regulation when the number is positive or down-regulation when it is negative). In A, changes in expression are expressed *vs.* shC vehicle-treated cells (set at 1 for each gene). In B, changes are expressed *vs.* the corresponding vehicle-treated cells (set at 1 for each gene).
